# Evaluation of a Behavioral Mobile Phone App Intervention for the Self-Management of Type 2 Diabetes: Randomized Controlled Trial Protocol

**DOI:** 10.2196/resprot.5959

**Published:** 2016-08-19

**Authors:** Shivani Goyal, Gary Lewis, Catherine Yu, Michael Rotondi, Emily Seto, Joseph A Cafazzo

**Affiliations:** ^1^ Centre for Global eHealth Innovation Techna Institute University Health Network Toronto, ON Canada; ^2^ Institute of Biomaterials and Biomedical Engineering University of Toronto Toronto, ON Canada; ^3^ Departments of Medicine and Physiology Division of Endocrinology and the Banting and Best Diabetes Centre University of Toronto Toronto, ON Canada; ^4^ Division of Endocrinology & Metabolism St. Michael's Hospital Toronto, ON Canada; ^5^ Li Ka Shing Knowledge Institute Division of Endocrinology and Metabolism St. Michael's Hospital Toronto, ON Canada; ^6^ School of Kinesiology and Health Science York University Toronto, ON Canada; ^7^ Institute of Health Policy, Management and Evaluation Dalla Lana School of Public Health University of Toronto Toronto, ON Canada

**Keywords:** diabetes mellitus, type 2, telemedicine, evaluation, self-care, randomized controlled trial, mobile applications, motivation, blood glucose

## Abstract

**Background:**

Patients with type 2 diabetes mellitus (T2DM) struggle with the management of their condition due to difficulty relating lifestyle behaviors with glycemic control. While self-monitoring of blood glucose (SMBG) has proven to be effective for those treated with insulin, it has been shown to be less beneficial for those only treated with oral medications or lifestyle modification. We hypothesized that the effective self-management of non-insulin treated T2DM requires a behavioral intervention that empowers patients with the ability to self-monitor, understand the impact of lifestyle behaviors on glycemic control, and adjust their self-care based on contextualized SMBG data.

**Objective:**

The primary objective of this randomized controlled trial (RCT) is to determine the impact of *bant2*, an evidence-based, patient-centered, behavioral mobile app intervention, on the self-management of T2DM. Our second postulation is that automated feedback delivered through the mobile app will be as effective, less resource intensive, and more scalable than interventions involving additional health care provider feedback.

**Methods:**

This study is a 12-month, prospective, multicenter RCT in which 150 participants will be randomly assigned to one of two groups: the control group will receive current standard of care, and the intervention group will receive the mobile phone app system in addition to standard of care. The primary outcome measure is change in glycated hemoglobin A1c from baseline to 12 months.

**Results:**

The first patient was enrolled on July 28, 2015, and we anticipate completing this study by September, 2018.

**Conclusions:**

This RCT is one of the first to evaluate an evidence-based mobile app that focuses on facilitating lifestyle behavior change driven by contextualized and structured SMBG. The results of this trial will provide insights regarding the usage of mobile tools and consumer-grade devices for diabetes self-care, the economic model of using incentives to motivate behavior change, and the consumption of test strips when following a rigorously structured approach for SMBG.

**Trial Registration:**

ClinicalTrials.gov NCT02370719; https://clinicaltrials.gov/ct2/show/NCT02370719 (Archived at http://www.webcitation.org/6jpyjfVRs)

## Introduction

### Background

The increasing global prevalence of diabetes is challenging traditional approaches towards diabetes management. It is not sustainable, nor cost effective, to assume that there will be an increase in health care delivery resources to address the growing prevalence of diabetes. Novel self-management tools, which aim to engage patients in daily diabetes care and optimize the role of health care professionals, may facilitate a more robust, scalable, and effective approach to the management of diabetes [1].

While self-management is a major component of chronic disease management, the majority of patients are not provided, or do not have access to, the tools and personalized education needed to engage in daily self-care practices [2] *.* Self-monitoring of blood glucose (SMBG) continues to be prescribed to patients as a self-management tool without the additional context, education, and frequent feedback required to interpret trends and adjust behaviors accordingly [3,4].

Furthermore, recent policy changes have reduced the reimbursements for test strips for patients who are not treated with insulin, further limiting access to SMBG as a self-monitoring tool [3]. These policy changes have been informed by evidence that evaluated SMBG as a standalone intervention, without the training required to understand the results of SMBG or frequent feedback from health care providers (HCPs). As such, patients that are not treated with insulin have limited support for test strip reimbursements, and also lack the education and tools needed to interpret and derive actionable knowledge from SMBG data.

Recent evidence has shown that when SMBG is performed in a structured manner, and is coupled with appropriate feedback and education, it may lead to positive health behavior change amongst patients with type 2 diabetes mellitus (T2DM) who are not treated with insulin [3]. As such, we hypothesized that the effective self-management of non-insulin treated T2DM requires a behavioral intervention that empowers patients with the ability to self-monitor, understand the impact of lifestyle behaviors on glycemic control, and adjust their self-care based on contextualized data.

The popularity of smartphones has presented an opportunity to deliver this type of intervention in the form of an evidence-based and user-centered diabetes self-management app [5-8]. The systematic design of the mobile health (mHealth) app *bant2* was informed by evidence and feedback from end-users, and is following a rigorous evaluation framework [1]. The evaluation framework is a blended model of the Knowledge to Action and Medical Research Council’s framework for complex interventions, and ensures that the intervention and study design, as well as the evaluation, follow a robust methodological approach [9].

The primary objective of this randomized controlled trial (RCT) is to determine the impact of the evidence-based, patient-centered, mobile app for the self-management of T2DM. Although there are many diabetes-focused electronic health (eHealth) tools currently available, the majority require a third party HCP to facilitate decision making [5]. Our second postulation is that automated feedback delivered through the mobile app will be as effective, less resource intensive, and more scalable than interventions involving additional HCP feedback.

## Methods

### Trial Design

The *bant2* study is a 12-month, prospective, multicenter, unblinded, parallel RCT in which 150 participants are randomly assigned to one of two groups: the control group receives current standard of care, and the intervention group receives the mobile phone app system in addition to standard of care. During the study period, all participants (intervention and control) attend quarterly clinic visits (standard care) and receive usual care.

The primary recruitment strategy for this study is through self-referral at primary care and community practices throughout the Greater Toronto Area. This area includes sites that are situated in urban and suburban settings. The University Health Network Research Ethics Board (REB) approved the study (14-7978-AE). Local institutional REB approvals were also obtained from the following participating sites: St. Joseph’s Health Care Centre (#2015-010), Trillium Health Partners (#698), and North York General (#15-0038). For sites without an institutational REB, namely the Taddle Creek Diabetes Education Program and LMC Diabetes and Endocrinology, approval was obtained from an Institutional Review Board (Pro00016415).

Randomization is being conducted at the patient level, using random block sizes of four and six to reduce the variance across the entire sample, with a 1:1 allocation ratio. Stratificaiton is also conducted by recruitment site. The allocation envelopes contain a piece of paper with either *control* or *intervention* written upon it, as well as a random patient identifier. The study coordinator allocates each participant by taking the envelope on the top of the stack, and enrolls the participant into the appropriate study arm, as indicated.

### Intervention

The participants randomly assigned to the intervention group will receive an iPhone 5S loaded with the *bant2* app, as well as a Bluetooth-enabled (a standard protocol for short distance wireless communication) Wahoo weight scale (Wahoo Fitness, Atlanta, GA, USA), a Jawbone UP24 (Jawbone, San Francisco, CA, USA) wrist-worn activity monitor, and a Jazz blood glucose meter (Agamatrix, Salem, NH, USA) [9]. We will also provide BlugluLe, a Bluetooth adapter that connects to the glucose meter and enables the wireless transfer of readings from the meter to the iPhone. The *bant2* mobile app also enables users to capture meal photos using the built-in camera, and track medication adherence on a weekly basis. In addition to the wrist-worn activity monitor, steps could also be tracked through the mobile phone itself using the built in accelerometer.

**Figure 1:**
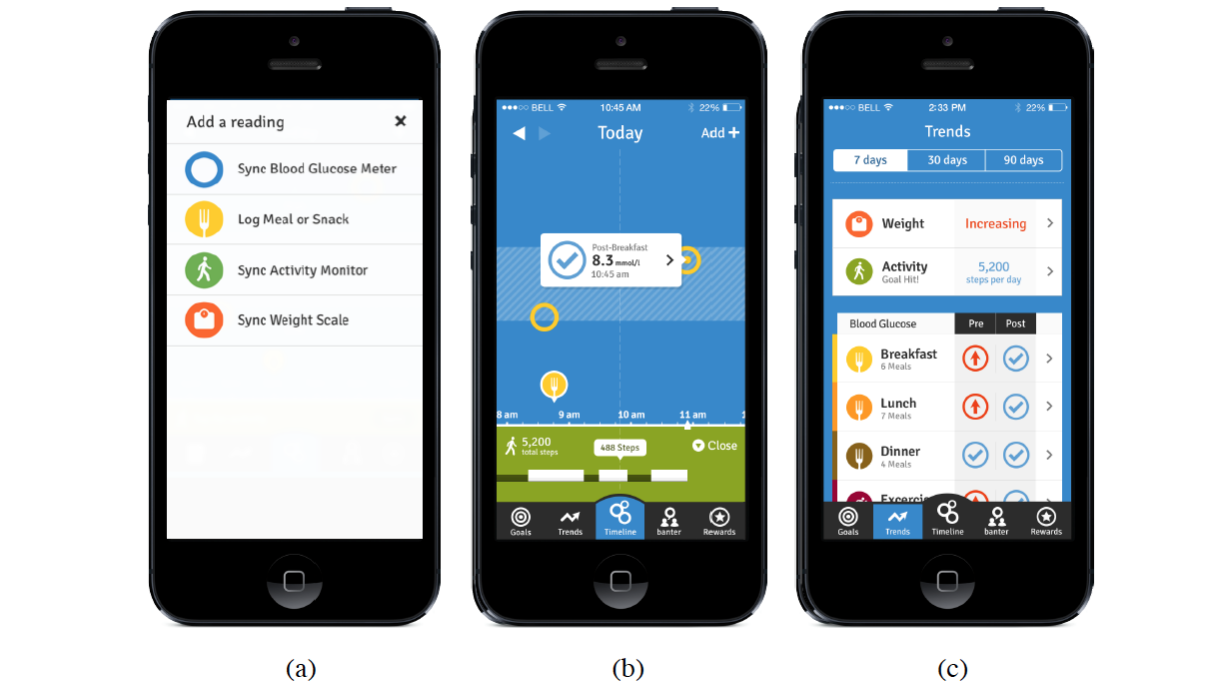
The bant2 mobile app enables users to monitor lifestyle behaviors and correlate them to their overall glycemic control.

The *bant2* app, shown in Figure 1, facilitates self-monitoring of lifestyle behaviors, and enables patients to correlate their lifestyle behaviors with their glycemic control through paired (pre- and post-prandial) blood glucose testing [10]. Upon data capture, the app will assess the other data points in context, identify positive and negative behaviours based on the analysis, and faciliate remedial decision making. The app also enables patients to set goals and receive reminders, participate in a closed-gated social community, and accumulate points for positive behaviors, which can be redeemed for tangible gift cards (eg, groceries, gym memberships) [9]. This app builds on a previous version of the *bant* app, which focused on engaging adolescents in the self-management of type 1 diabetes mellitus (T1DM). A 12-week pilot of *bant* app demonstrated positive behavior change amongst the adolescents with T1DM [11].

We have ensured that all of the appropriate safeguards are in place to protect personal health information. For example, the iPhone requires a passcode to access the app, and the app itself requires a username and password. In the event that the device is lost or stolen, we can remotely wipe the device using mobile data management software (Airwatch, Atlanta, GA). The *bant2* app is intended to provide guidance and facilitate diabetes self-management, and is not classified as a medical device. Given that the app poses minimal risk to the patient, Health Insurance Portability and Accountability Act requirements do not apply.

### Participants

The inclusion criteria for participants include: definitive diagnosis of T2DM, not treated with insulin, at least 18 years of age, a baseline glycated hemoglobin A1c (A1c) of 7.5% or higher, and ability to speak and read English.

Exclusion criteria include: inability to use a mobile phone (eg, due to vision problems) or to comply with home monitoring (eg, due to suffering from anxiety or depression), and duration of diabetes under one year. Participants who have had diabetes for less than one year are excluded because they typically demonstrate a higher adherence to self-care and have high levels of motivation [12].

### Outcomes

The primary outcome measure is change in A1c from baseline to 12 months. Each recruitment site is provided with a DCA Vantage Analyzer (Siemens, Munich, Germany) point of care A1c device to reduce the variability in the A1c laboratory assays. As secondary end points, A1c measurements are also collected at 3-month intervals (3, 6, and 9 months). Clinical staff within the patient’s circle of care are conducting the point of care A1c tests.

Secondary outcomes include blood pressure (mmHg), weight (pounds), total cholesterol (mmol/L), Low Density Lipoprotein cholesterol (mmol/L), and weight at baseline, 3, 6, 9, and 12 months. The number of participants who achieve optimal glycemic control (A1c <7%), as well as the type and frequency of medication changes, will also be measured. Furthermore, validated instruments described in Table 1 are being used to collect and measure burden of disease and diabetes-related self-efficacy and self-care, pre-, mid-, and post-study.

**Table 1:** Validated instruments administered pre-, mid-, and post-study.

Measure	Description
Diabetes Distress Scale (DDS)	The DDS is a 17-item instrument that assesses the emotional, physician-related, regimen-related, and interpersonal aspects of diabetes distress, and provides an indication of diabetes-related quality of life [13].
Diabetes Empowerment Scale - Short Form (DES-SF)	The DES-SF is a 28-item instrument used to measure psychosocial self-efficacy of diabetes self-management. This tool focuses on (1) managing diabetes, (2) assessing readiness to change, and (3) willingness to set goals and change behaviors [14,15].
Summary of Diabetes Self-Care Activities (SDSCA) measure	The SDSCA is a 11-item instrument that assesses individual levels of diabetes self-care, focusing on general diet, specific diet, exercise, medication adherence, blood-glucose testing, smoking, and foot care [16].
The Mobile Application Rating Scale (MARS) App - User Version	The MARS is a 26-item instrument used to evaluate the quality, functionality, and overall satisfaction of the mobile app itself [17].

### Sample Size

The sample size estimation was based on detecting a minimum reduction of 0.5% in A1c values. Given our inclusion criteria of A1c >7.5%, we anticipate that A1c levels will be highly clustered around 8.5% (standard deviation 1.0). A minimum of 63 participants per group is necessary to detect a difference of 0.5% in A1c, at an 80% power with a one-sided 5% significance level in all anticipated cases. With a further 15% adjustment for potential dropouts, a final sample size of 150 subjects will be required for both groups, with 75 participants in each study arm. All calculations were performed using the R software package, *epibasix* [18].

### Recruitment Procedure

#### Physician Recruitment

The lead physician (or research administration staff) of the recruitment site is first approached by email regarding their interest in the study, and a 20-30 minute presentation is offered as a way to disseminate the study details and gauge interest across the site. If the lead physician is interested in participating, they contact the clinicians within their site, disseminate information regarding the study, and identify the clinicians interested in participating in the study. Upon receiving REB approval, a staff member at the participating site generates a list of eligible participants and has the list vetted by the respective physicians for appropriateness.

#### Patient Recruitment

The participating sites send invitation letters to eligible and appropriate participants, explaning the study goals and protocol. The letter invites participants to contact the study coordinator directly, either by email or phone call, if they are interested in participating in the study. Upon initial contact, the study coordinator describes the study and answers any of the patient’s study-related questions. If the respondent is interested in participating, the coordinator will schedule an appointment with the respective clinic, and email or mail the patient the consent form in advance for review.

### Data Analyses

The principal analysis strategy will be the use of linear mixed models in the Statistical Analysis System (Cary, NC, USA) using the PROC MIXED procedure. This approach provides a simple method to incorporate baseline values and the correlation of each participant over time (using a random effect). This model is more powerful than the repeated measures *analysis of variance*, as it can easily examine differences between the RCT groups at all time points, and can accommodate potential confounders. Furthermore, although the A1c measurements will be made at 3-month intervals, our primary analysis will focus on A1c levels at the conclusion of the 12-month study. A contrast will be constructed to test this primary comparison among all possible pair-wise tests; no adjustments for multiple testing will be required. Subsequent adjustments will be made for potential confounding variables as necessary, such as baseline parameters that may vary between groups (eg, age, sex).

Secondary contrasts will be used to examine differences from baseline for all other time points; however, these will be of exploratory interest only. Moreover, we will use regression analysis to separate the contribution of the different intervention components (activity monitor, weight scale, SMBG, mobile app features), and determine their independent effects on the primary outcome at 12 months. This approach will identify which aspect of the mHealth intervention contributed to the change in A1c.

## Results

This trial is currently open for recruitment. The anticipated completion date for the study is September, 2018.

## Discussion

SMBG is an essential part of managing glycemic control. However, for T2DM patients that are not treated with insulin, the standard approach of simply recommending SMBG without the appropriate guidelines and training will not facilitate behavior change [4]. Polonsky outlines four main considerations for SMBG amongst this specific population: (1) SMBG should be structured and performed regularly around key events (eg, meals), (2) patients need to be provided with SMBG-related training, (3) clinicians must be able to view SMBG data and use it to inform clinical decisions, and (4) useful display of SMBG data to facilitate pattern identification [3]. To our knowledge, the *bant2* app is the first mobile app to facilitate structured SMBG, enable simple pattern and trend detection, and potentially facilitate communication between the patient and HCPs during clinic visits.

This RCT will evaluate the use of the *bant2* app as a self-management tool compared to standard care, over a period of 12 months. We anticipate that the use of the app will provide patients with a greater understanding of which aspects of lifestyle behaviors impact glycemic control, increase participation in self-care activities, and potentially improve diabetes outcomes. We also anticipate that along with higher levels of engagement, patients will initiate conversations with their HCPs, using the SMBG summary data displayed in the app as a reference during consultations. The sharing of such data may result in earlier treatment optimization and medication changes in the intervention group compared to the control group.

A considerable limitation of the study is that the iPhone provided to the intervention group is likely to be a secondary device, potentially hindering the complete immersion of the app into daily routines.

At the time of the intervention design, there were no Bluetooth-enabled blood glucose meters available in Canada. In order to facilitate the wireless transfer of blood glucose readings to the app, and reduce burden and errors associated with manual entry, we had to develop a customized Bluetooth adapter [9]. Future studies should explore how apps can be installed directly on an individual’s personal devices, and explore ways to utilize off-the-shelf meters that consumers are already familiar with (and which meters are potentially reimbursable). These issues highlight the challenges of RCTs as an evaluation approach for mobile apps. Given the rapid evolution of technology (eg, Bluetooth-supported devices), the mHealth interventions evaluated in trials are often no longer relevant once the lengthy trials have concluded several years later [19].

Furthermore, as outlined by Polonsky [3], we were not able to provide additional education and skills training to providers. However, we anticipate that through the use of *bant2*, patients will develop an understanding of glycemic control and self-management skills, leading to improved lifestyle management.

The results of this study will further our understanding of how an evidence-based behavior modification mobile app can guide patients in the self-management of their diabetes. Specifically, we will be able to assess how various features of the *bant2* app influence self-management behaviors. For example, we will examine how consumer-grade devices (such as wearable devices) facilitate chronic disease management, and how incentive mechanisms potentially motivate behavior change. Most importantly, the study data will demonstrate how test strips are consumed when SMBG is performed in a systematic way, and the impact of immediate feedback on glycemic control and trends.

### Conclusions

This RCT is one of the first studies to evaluate an evidence-based mobile app that focuses on facilitating lifestyle behavior change driven by contextualized and structured SMBG. The results of this trial will provide insights regarding the usage of mobile tools for diabetes self-care, the effectiveness of consumer-grade devices for chronic disease management, the economic model of using incentives to motivate behavior change, and the consumption of test strips when SMBG follows a rigorously structured approach. The findings from this study will inform the next generation of diabetes education, guidelines for SMBG, and potentially inform health policy pertaining to strip reimbursements for T2DM patients that are not treated with insulin.

## Abbreviations

A1c: glycated hemoglobin A1c
DDS: Diabetes Distress Scale
DES-SF: Diabetes Empowerment Scale - Short Form
HCP: health care provider
MARS: Mobile Application Rating Scale
mHealth: mobile health
RCT: randomized controlled trial
REB: Research Ethics Board
SDSCA: Summary of Diabetes Self-Care Activities
SMBG: self-monitoring of blood glucose
T1DM: type 1 diabetes mellitus
T2DM: type 2 diabetes mellitus
